# Evaluation of the middle-range theory of occupational stress in healthcare professionals

**DOI:** 10.1590/0034-7167-2024-0505

**Published:** 2025-11-03

**Authors:** Romanniny Hévillyn Silva Costa Almino, Richardson Augusto Rosendo da Silva, Larissa Lilian Costa Firmino Segundo, Yasmim Yngrid Fernandes de Freitas, Vinicius dos Santos Lemos Pereira, Isabele Silva dos Santos, Thais Targino Ferreira, Roberta Keile Gomes de Sousa Manso

**Affiliations:** I Empresa Brazileira de Serviços Hospitalares. Natal, Rio Grande do Norte, Brazil; II Instituto Federal do Rio Grande do Norte. Natal, Rio Grande do Norte, Brazil; III Universidade Federal do Rio Grande do Norte. Natal, Rio Grande do Norte, Brazil

**Keywords:** Occupational Stress, Health Personnel, Nursing, Validation Study, Nursing Theory, Estrés Laboral, Personal de Salud, Enfermería, Estudio de Validación, Teoría de Enfermería

## Abstract

**Objectives:**

to evaluate the Middle-Range Theory of Occupational Stress in Healthcare Professionals based on the opinions of expert judges.

**Methods:**

this is a methodological study. The theory was evaluated using Fawcett’s criteria, and the phases described by Borel et al. were followed for the evaluation of nursing theories.

**Results:**

the evaluation was conducted in two rounds. The sample consisted of five judges. In the first round, the theory was considered adequate regarding significance, parsimony, testability, and empirical adequacy. However, one item related to internal consistency and one item related to pragmatic adequacy were not considered adequate. In the second round, the previously unvalidated items were deemed adequate.

**Conclusions:**

the Middle-Range Theory of Occupational Stress in Healthcare Professionals was evaluated as adequate, and the alternative hypothesis was accepted. The theory will allow for the identification of stressors and protective factors related to occupational stress, as well as the implementation of interventions by nurses, demonstrating the impact of the study.

## RESUMEN

**Objetivos:**

evaluar la Teoría de Alcance Medio del Estrés Ocupacional en Profesionales de la Salud con base en la opinión de jueces.

**Métodos:**

se trata de un estudio metodológico. La evaluación de dicha teoría se realizó a partir de los criterios de Fawcett y se utilizaron las fases descritas por Borel et al. para la evaluación de teorías de enfermería.

**Resultados:**

la evaluación se llevó a cabo en dos rondas. La muestra estuvo compuesta por cinco jueces. En la primera ronda, la teoría fue considerada adecuada en cuanto a significación, parsimonia, verificabilidad y adecuación empírica. Sin embargo, un ítem relacionado con la consistencia interna y un ítem relacionado con la adecuación pragmática no fueron considerados adecuados. En la segunda ronda, los ítems no validados previamente fueron considerados adecuados.

**Conclusiones:**

la Teoría de Alcance Medio del Estrés Ocupacional en Profesionales de la Salud fue evaluada como adecuada, aceptándose la hipótesis alternativa. La teoría permitirá la identificación de los estresores, de los factores de protección relacionados con el estrés ocupacional y la realización de intervenciones por parte del enfermero, demostrando el impacto del estudio.

## INTRODUCTION

The development of nursing theories plays a fundamental role in advancing practice, research, and education in the field. These theories provide an essential framework for professional practice, guiding decision-making and contributing to the improvement of care quality delivered to patients^(1)^. Furthermore, they enable a deeper understanding of phenomena related to health and care, facilitating communication among healthcare professionals and promoting standardization of practices^(2)^.

Among the various theoretical approaches, Middle-Range Theories (MRT) stand out for their applicability in healthcare settings. Unlike grand theories, which tend to be more abstract and comprehensive, MRTs are more specific, focusing on particular aspects of care, making them highly relevant for daily clinical practice^(3)^. A particularly significant application of MRTs is in occupational health care, especially among healthcare professionals.

These professionals are constantly exposed to various stressors in the workplace, including excessive workloads, high emotional demands, and the risk of exposure to infectious diseases. Such factors can lead to high levels of stress, burnout, and deterioration of mental and physical health^(4)^. Therefore, attention to the health of these workers is essential, not only for their individual well-being but also to ensure the quality of care provided to patients.

The application of nursing theories in occupational health care allows for understanding and addressing the factors impacting professionals’ well-being, such as occupational stress^(5)^. Within this context, several nursing theories have been developed, including Betty Neuman’s Systems Model, which can generate an MRT through a deductive process. According to this model, the patient (client) interacts constantly with their environment and is subject to stressors that can disrupt system stability, triggering stress responses, including the development of diseases. Consequently, nurses play a crucial role in implementing strategic interventions to restore the client’s system balance^(6)^.

A study proposed the Occupational Stress Nursing Diagnosis, developed from the Middle-Range Theory of Occupational Stress in Healthcare Professionals (MRT-EOPS)^(7)^. This theory was based on conceptual analysis, drawing from the theoretical framework of Walker and Avant^(8)^ and operationalized through a scoping review derived from Betty Neuman’s Systems Model^(6)^.

The MRT-EOPS was constructed deductively from Betty Neuman’s Systems Model, incorporating key concepts such as system, client, intra-, inter-, and extrapersonal stressors, client system instability, disease, reactions, interaction variables, client system stability, well-being/health, nursing, and primary, secondary, and tertiary prevention. This theory positions healthcare professionals at the core, considering their susceptibility to intra-, inter-, and extrapersonal stressors, as well as protective factors. If the healthcare professional exhibits negative responses to stressors, they may develop instability, manifested through physiological and psychological symptoms, ultimately leading to occupational stress^(7)^.

Furthermore, the evaluation of theories enables assessing their relevance and adequacy for research, education, clinical practice, and nursing management^(9,10)^, thus justifying the current study.

Given this scenario and the importance of evaluating the developed theory, the following research question emerges: Does the MRT-EOPS meet the criteria of significance, internal consistency, parsimony, testability, empirical adequacy, and practicality?

## OBJECTIVES

To evaluate the Middle-Range Theory of Occupational Stress in Healthcare Professionals (MRT-OSHCP) based on the opinions of expert judges, using Fawcett’s criteria^(11)^ and the phases described by Borel et al^(12)^.

## METHODS

### Ethical aspects

This research adhered to the ethical principles established by Resolution 466/12 of the National Health Council and was approved by the Research Ethics Committee.

### Study design, period, and setting

This study is a methodological investigation aimed at evaluating the Middle-Range Theory of Occupational Stress in Healthcare Professionals (MRT-EOPS). To conduct the evaluation, the Delphi method was employed, based on the criteria established by Fawcett^(11)^. The Delphi method is widely utilized to obtain collective consensus on a given topic assessed by a panel of experts^(12,13)^.

In this study, following the evaluation of a nursing theory, the phases outlined by Borel et al^(12)^ were adopted: Preparatory phase, Intermediate phase and Theory evaluation phase. The evaluation phase was conducted from June to November 2022.

The preparatory phase consisted of defining the theory to be analyzed and selecting the evaluation criteria. Accordingly, the MRT-EOPS was assessed based on the following criteria proposed by Fawcett^(11)^: Significance, Internal consistency, Parsimony, Testability, Empirical adequacy and Pragmatic applicability.

Only one adaptation was made in the criterion of internal consistency, with the inclusion of the following evaluation aspect: *“the diagram/pictogram demonstrates structural consistency”*. Additionally, this research was structured in accordance with the guidelines of the Revised Standards for Quality Improvement Reporting Excellence (SQUIRE 2.0)^(14)^.

### Population or sample; inclusion and exclusion criteria

The selection of evaluators was conducted through a search on the Lattes Platform, maintained by the National Council for Scientific and Technological Development (CNPq), using the following keywords: “nursing” AND “nursing theory” AND “validation study”.

Subsequently, the evaluators of MRT-EOPS were categorized according to the criteria proposed by Borel et al^(12)^, considering: Educational background, Professional experience, Metatheoretical knowledge, Dissemination of research in the field and Recognition of expertise in metatheories or nursing theories.

It is noteworthy that, for the criterion “professional experience in the field of the theory under evaluation”, general experience in nursing theories was considered, given that the MRT-EOPS was still in its development phase.

The evaluators were invited via email, through a formal invitation letter containing the Informed Consent Form (ICF), which included detailed information about the study and instructions for completing the evaluation instrument. The email addresses were obtained from the Lattes Platform, institutional websites, or other online sources. An initial deadline of 20 days was established for responses from evaluators who agreed to participate in the study. Additionally, periodic reminders were sent to the selected evaluators every 15 days to encourage participation.

### Study protocol

The following section details the nine key points that compose the intermediate phase, as outlined by Borel et al^(12)^:

1) Definition of the Delphi Method Type: A Likert scale-based evaluation instrument was utilized, incorporating spaces for suggestions and comments from the evaluators.
2) Roles of the Coordinator, Primary Evaluator, and Secondary Evaluators: The coordinator/primary evaluator was responsible for providing secondary evaluators with the necessary materials for the MRT-EOPS assessment^(12)^.
3) Selection of Evaluator Candidates: According to Borel et al^(12)^, judges were classified based on their presumed level of expertise, categorized as novice, advanced novice, competent, proficient, or expert. It is noteworthy that sample size depends on the evaluators’ level of expertise.
4) Invitation to Evaluators: As previously mentioned, invitations were sent via email, including the Informed Consent Form (ICF).

**Figure 1:**
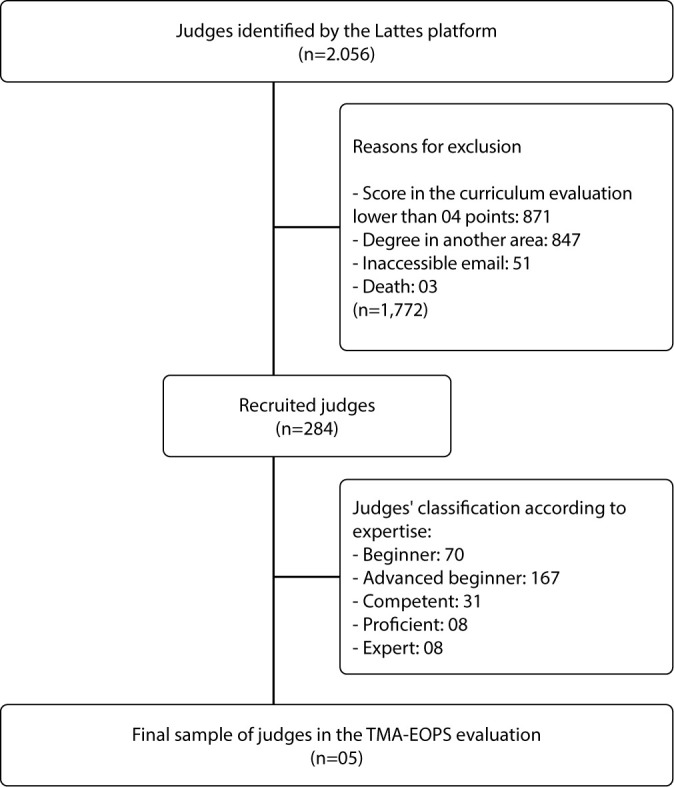
Flowchart of the Judge Recruitment Process for the Evaluation of the Middle-Range Theory of Occupational Stress in Healthcare Professionals (MRT-EOPS)

In Figure 1, there is the flowchart depicting the recruitment process of judges for the evaluation of MRT-EOPS.

5) Reapplication of the Evaluator Categorization Criteria Based on Expertise Level and Adjustments to Judge Teams: After the initial categorization, no adjustments were necessary in the composition of evaluator teams.
6) Planning of Evaluation Rounds and Interruption Criteria: The number of rounds was determined based on the achievement of consensus among the judges. Two rounds of evaluation were conducted, and the minimum cutoff threshold for reaching the predefined agreement level in this study was 0.8 or 80%^(15)^.
7) Establishment of Explicit Criteria for Item Exclusion in Each Round: Items that did not meet the minimum agreement threshold were excluded from subsequent analyses.
8) Definition of Consensus Achievement and Stability of Judge Responses: The evaluation of items was conducted using a five-point Likert scale, with the following options: 1 – Strongly disagree, 2 – Partially disagree, 3 – Neutral (neither agree nor disagree), 4 – Partially agree and 5 – Strongly agree. For data analysis, all variables measured on the Likert scale were grouped into three categories: 1 and 2 – Disagreement, 3 – Neutrality and 4 and 5 – Agreement.
9) Provision of Guidelines on the Theoretical Evaluation Strategy to Judges: Judges received detailed instructions on theoretical evaluation criteria, ensuring greater clarity and uniformity in the analysis of proposed items.

### Analysis of results and statistics

The proposed methods were carried out using Microsoft Excel 2019 and R software, version 4.2.2. Initially, a descriptive and inferential analysis of the data was conducted. The statistical tests employed in this study included: Content Validity Index (CVI), Aiken’s V coeficiente and Free marginal multi-evaluator Kappa.

Additionally, the following statistical hypotheses were established in relation to the evaluation of the Middle-Range Theory of Occupational Stress in Healthcare Professionals (MRT-EOPS): Null hypothesis (H₀): The items of MRT-EOPS evaluated as adequate exhibit an index below 80%. Alternative hypothesis (H₁): The items of MRT-EOPS evaluated as adequate exhibit an index equal to or greater than 80%.

## RESULTS

It was observed that the majority of judges were female (60%), held a doctoral degree (80%), and worked as faculty members (80%). Additionally, 40% of the evaluators were classified as experts, demonstrating a high level of specialization in the field. Moreover, 80% of the participants were engaged in activities related to mentorship and studies on nursing theories.

Regarding the Content Validity Index (CVI), Aiken’s V coefficient, and the free marginal multi-evaluator Kappa, it was found that only the item “Possibility of comparing the results obtained with the implementation of nursing interventions derived from MRT and without the use of the theory” did not reach the critical threshold of 0.8, making its reassessment necessary, as shown in Table 1.

**Table 1:** Evaluation by judges, in the second round, of the Middle-Range Theory of Occupational Stress in Healthcare Professionals, according to Fawcett’s criteria, based on the CVI, Aiken’s coefficient, and free-marginal multirater Kappa, Natal, Rio Grande do Norte, Brazil, 2022

CRITERION	ITEM	CVI	Aiken's Coefficient	Kappa
Significance	The concepts and propositions of the MRT are explicit, including the metaparadigmatic concepts.	1.00	1.00	1.00
	The philosophical statements on which the theory is based are explicit.	1.00	1.00	1.00
	The conceptual model from which the MRT was derived is explicit.	1.00	1.00	1.00
Internal consistency	There is congruence between the context (philosophical aspects and conceptual model) and the content (concepts and propositions) of the MRT.	1.00	1.00	1.00
	The concepts demonstrate clarity and semantic consistency.	1.00	1.00	1.00
	The propositions demonstrate semantic consistency.	1.00	1.00	1.00
	The propositions demonstrate structural consistency.	1.00	1.00	1.00
	The diagram/pictogram demonstrates structural consistency.	1.00	1.00	1.00
Parsimony	The theoretical content is stated clearly and concisely.	1.00	1.00	1.00
Testability	The research methodology reflects the Middle-Range Theory.	1.00	1.00	1.00
	The concepts of the Middle-Range Theory are observable through appropriate empirical indicators.	1.00	1.00	1.00
	The theoretical propositions are subject to empirical testing.	1.00	1.00	1.00
Empirical adequacy	The theoretical statements are congruent with empirical evidence.	1.00	1.00	1.00
Pragmatic adequacy	The teaching of the MRT is necessary before applying the theory in nursing practice.	1.00	1.00	1.00
	The derivation of the MRT is feasible.	1.00	1.00	1.00
	Nurses have the legal authority to implement and measure the effectiveness of the theory based on nursing interventions.	1.00	1.00	1.00
	Nursing interventions based on the MRT are feasible for implementation in nursing practice.	1.00	1.00	1.00
	Nursing interventions based on the MRT may lead to favorable outcomes.	1.00	1.00	1.00
	It is possible to compare the results obtained with the implementation of nursing interventions derived from the MRT and without the use of the theory.	1.00	1.00	1.00
	The results can be measured in terms of the effectiveness of the MRT in solving problems.	1.00	1.00	1.00
	Global evaluation			1.00

*MRT – Middle-Range Theory; CVI – Content Validity Index.*

Additionally, the item “The diagram/pictogram demonstrates structural consistency” obtained an index below 0.8 in the free marginal multi-evaluator Kappa test, conducted in the first evaluation round, prompting recommendations for enhancements in the theoretical framework.

After the modifications suggested by the judges in the second evaluation round, the item “Possibility of comparing the results obtained with the implementation of nursing interventions derived from MRT and without the use of the theory” achieved the highest possible score in the following indicators: Content Validity Index (CVI), Aiken’s V coeficiente and Free marginal multi-evaluator Kappa. The detailed results are presented in Table 2.

**Table 2:** Evaluation by judges, in the second round, of the Medium-Range Theory of Occupational Stress in Health Professionals, according to Fawcett criteria, according to IVC, Aiken coefficient and free marginal multi-rater kappa, Natal, Rio Grande do Norte, Brazil, 2022

CRITERION	ITEM	CVI	Aiken's Coefficient	Kappa
Significance	The concepts and propositions of the TMA are explicit, including the metaparadigmatic concepts.	1.00	1.00	1.00
	The philosophical claims on which the theory is based are explicit.	1.00	1.00	1.00
	The conceptual model from which the TMA was derived is explicit.	1.00	1.00	1.00
Internal consistency	There is congruence between the context (philosophical aspects and conceptual model) and the content (concepts and propositions) of the TMA.	1.00	1.00	1.00
	The concepts are clear and semantically consistent.	1.00	1.00	1.00
	The propositions are semantically consistent.	1.00	1.00	1.00
	The propositions are structurally consistent.	1.00	1.00	1.00
	The diagram/pictogram is structurally consistent.	1.00	1.00	1.00
Parsimony	The theoretical content is stated clearly and concisely.	1.00	1.00	1.00
Testability	The research methodology reflects Middle-Range Theory.	1.00	1.00	1.00
	The concepts of Middle-Range Theory are observable through appropriate empirical indicators.	1.00	1.00	1.00
	The theoretical propositions are amenable to empirical testing.	1.00	1.00	1.00
Empirical adequacy	The theoretical claims are consistent with empirical evidence.	1.00	1.00	1.00
Pragmatic adequacy	Teaching of TMA is necessary before applying the theory to nursing practice.	1.00	1.00	1.00
	The derivation of TMA is feasible.	1.00	1.00	1.00
	The nurse has the legal capacity to implement and measure the effectiveness of theory-based nursing interventions.	1.00	1.00	1.00
	Nursing interventions based on TMA are compatible with implementation in nursing practice.	1.00	1.00	1.00
	Nursing interventions based on TMA may lead to the achievement of favorable outcomes.	1.00	1.00	1.00
	There is a possibility of comparing the results obtained with the implementation of nursing interventions derived from TMA and without the use of theory.	1.00	1.00	1.00
	The results may be measured in terms of the effectiveness of TMA in solving problems.	1.00	1.00	1.00
	Overall assessment.			1.00

TMA – Middle Range Theory; CVI – content validation index.

Based on the judges’ recommendations during the evaluation, the need for adjustments in the diagram of the Middle-Range Theory of Occupational Stress in Healthcare Professionals (MRT-EOPS) was identified. The key recommendations were as follows:

- Correction of the direction of stressor arrows, which previously pointed toward system stability in the health-disease process. An adjustment was made to the vector directions to better represent the influence of stressor factors.
- Modification of the diagram colors, aiming for greater clarity in distinguishing concepts related to the alleviation and emergence of occupational stress.
- Expansion of the explanation regarding the theory’s applicability, as suggested by one of the evaluators, to reinforce its practical application and scientific relevance.

Given these observations, adjustments to the diagram were implemented, based on principles of chromotherapy, as described below:

- Arrows originate from the center of the diagram, symbolizing the healthcare professional’s response to the influence of stressors and protective factors in their health-disease process.
- Blue: Represents concepts related to the stability of the health-disease process, including health and protective factors.
- Red: Indicates elements associated with instability in the health-disease process, encompassing stressors, physiological and psychological symptoms, and occupational stress.
- Green: Refers to concepts aimed at maintaining stability in the health-disease process, including nursing, nursing interventions, health promotion, and secondary and tertiary prevention.

**Figure 2:**
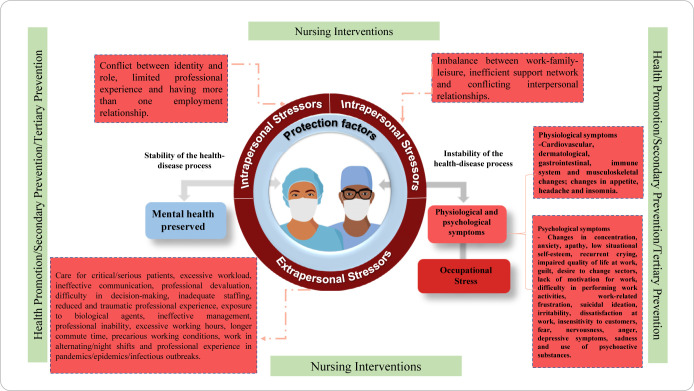
Diagram of the Middle-Range Theory of Occupational Stress in Healthcare Professionals (MRT-EOPS)

The implemented modifications are visually represented in Figure 2.

Therefore, after the modifications suggested by the judges in the second round of evaluation, the item “the diagram/pictogram demonstrates structural consistency” achieved the maximum score for the Content Validity Index (IVC), Aiken’s coefficient, and Multi-Rater Kappa.

Thus, since the IVC for all evaluated items was greater than 0.8, the critical Aiken’s value was ≥ 0.8, and/or the marginal free Multi-Rater Kappa exceeded 0.8, the alternative hypothesis was accepted in the study.

Considering that the Middle-Range Theory of Occupational Stress in Healthcare Professionals (MRT-EOPS) was recently developed, there has not yet been an opportunity to compare data on the implementation of nursing interventions derived from the theory with those obtained without its use.

Given this limitation, the importance of testing the MRT-EOPS is highlighted to evaluate its scientific credibility and practical applicability. To this end, it is recommended that future studies employ different methodological approaches, including qualitative, quantitative, and mixed-method designs, to test the theory across various nursing practice domains, such as clinical care, education, research, and management.

Following the modifications suggested by the judges during the second evaluation round, the item “The diagram/pictogram demonstrates structural consistency” achieved the highest possible score in the following indicators: Content Validity Index (CVI), Aiken’s V coeficiente and Free marginal multi-evaluator Kappa.

Furthermore, since the CVI scores for all evaluated items were above 0.8, the critical value for Aiken’s V coefficient was ≥ 0.8, and the free marginal multi-evaluator Kappa results exceeded 0.8, the alternative hypothesis was accepted in the study.

## DISCUSSION

Nursing theories provide care models that enhance nurses’ clinical practice^(16)^. In other words, they establish the scientific foundation of knowledge and contribute to the refinement of professional activities through the planning and organization of care processes.

In this context, Middle-Range Theories (MRTs) represent effective methods for bridging theory and practice, serving as structuring references for nursing clinical practice^(17)^. The evaluation of MRTs, particularly during their development phase, enables the determination of the theory’s relevance across various nursing domains, while also ensuring compliance with established assessment criteria^(10,11)^.

The Fawcett Method comprises a set of specific criteria aimed at evaluating nursing theories based on significance, internal consistency, parsimony, testability, empirical adequacy, and pragmatic utility^(11)^. Thus, the Fawcett Method was selected and employed as a strategy to examine the relevance of MRT-EOPS during the preparatory phase of the Middle-Range Theory, following the Delphi method, as outlined by Borel et al^(12)^.

The criteria established by Borel et al^(12)^ were applied in selecting the experts responsible for assessing the Middle-Range Theory of Occupational Stress in Healthcare Professionals (MRT-EOPS). These criteria encompass essential aspects for classifying the evaluators’ level of expertise, including educational background, professional experience, metatheoretical knowledge, dissemination of knowledge produced in the field, and recognition of expertise in metatheories or nursing theories.

The MRT-EOPS developed in this study was assessed by experts using the Delphi method, which relies on collective wisdom. Theory evaluation plays a crucial role in the advancement of nursing knowledge, as it facilitates the refinement of its components—enhancing them prior to dissemination in practice and following their application^(10,12)^.

In the first phase of MRT-EOPS evaluation, the experts deemed the following criteria appropriate: significance, parsimony, testability, and empirical adequacy.

According to Fawcett^(11)^, the significance of a theory encompasses its metaparadigmatic and philosophical origins, as well as the conceptual models from which it was derived. It is essential that such information be clearly articulated.

The MRT-EOPS was derived from Betty Neuman’s theoretical model, which adopts a holistic philosophical perspective and is influenced by General Systems Theory, establishing direct connections with Gestalt concepts and stress theory. Moreover, this theory is oriented toward the promotion of well-being^(7)^.

Parsimony refers to the evaluation of a theory’s content, emphasizing simplicity and objectivity. A well-structured theory should present a limited number of concepts and propositions without compromising its explanatory capacity, ensuring sufficient clarity in elucidating the phenomenon of interest^(11)^. In the case of MRT-EOPS, 16 key concepts and 20 propositions were defined^(7)^.

The criterion of testability, in turn, is linked both to the content of the theory and its practical applicability. For a Middle-Range Theory (MRT) to be considered testable, its concepts must have operational definitions, while its propositions must be subject to observation and/or measurement^(11)^. In the context of MRT-EOPS, the definitions of stressors and symptoms addressed in the theory were explicitly described^(7)^.

According to Fawcett^(11)^, empirical adequacy is confirmed when the statements within the theory are grounded in scientific evidence. In the case of MRT-EOPS, all concepts and propositions were derived from the findings of the 138 publications that constituted the Scoping Review^(7)^.

Finally, internal consistency refers to the clarity and semantic coherence of the theory. All concepts, propositions, and statements must exhibit structural and logical congruence^(11)^. During the evaluation of MRT-EOPS, its initial graphical representation was found to lack complete consistency; however, after modifications suggested by the experts, this aspect was corrected and refined.

The presentation of the MRT-EOPS diagram reinforces the understanding of the theory, highlighting its two core components: stress and the response to stress. These elements are clearly and systematically arranged within the visual model, enabling healthcare professionals to better assimilate the concepts^(18)^.

The MRT-EOPS diagram incorporates principles of chromotherapy, a science applied in healthcare. The colors used hold therapeutic significance, serving to represent different aspects of the theory. Blue plays a crucial role in stress reduction, while green aids in stress relief and symbolizes the healthcare field. Meanwhile, red is associated with stress^(19)^.

By aligning theory with clinical practice, MRT-EOPS provides substantial benefits for both education and research, fostering greater integration between professional practice and academic knowledge. Thus, the theory strengthens the scientific foundation of occupational health nursing, offering essential guidelines for nurses’ professional practice.

Additionally, healthcare management is also addressed, as the theory emphasizes the importance of interdisciplinary collaboration in developing effective strategies for the prevention of occupational stress.

The practical applications of the theory involve the identification of intra-, inter-, and extrapersonal stressors, as well as protective factors against occupational stress. MRT-EOPS also guides the implementation of preventive interventions, which can be applied at primary, secondary, and tertiary levels.

Finally, MRT-EOPS adopts a holistic philosophical approach, providing a consistent explanation of the phenomenon of occupational stress in healthcare professionals. It offers a solid foundation for strategic and preventive actions in clinical settings.

### Study limitations

Considering that the Middle-Range Theory of Occupational Stress in Healthcare Professionals (MRT-EOPS) was recently developed, there has not yet been an opportunity to compare implementation data or the results of nursing interventions derived from the theory. This represents a limitation of the study.

Thus, the importance of future validations in clinical settings is highlighted, allowing for the practical application of the theory and an assessment of its effectiveness. To achieve this, it is recommended that research studies employ different methodological approaches, including qualitative, quantitative, and mixed-method designs, to test MRT-EOPS across various nursing practice domains, such as clinical care, education, research, and management.

### Study contributions to the fields of Nursing, Health, or Public Policy

The Middle-Range Theory of Occupational Stress in Healthcare Professionals (MRT-EOPS) presents itself as a promising tool for the clinical practice of nurses working in the occupational health sector, as it is grounded in an evidence-based approach.

Furthermore, it enables the identification of stressors and protective factors related to occupational stress, allowing for the implementation of effective interventions aimed at promoting workers’ mental health.

## CONCLUSIONS

This study enabled the assessment of the Middle-Range Theory of Occupational Stress in Healthcare Professionals (MRT-EOPS) based on Fawcett’s criteria, through the application of the Delphi method, following the phases outlined by Borel et al.

In the first evaluation round, two items presented indices below the expected threshold, necessitating adjustments and revisions: “Possibility of comparing the results obtained with and without the implementation of nursing interventions based on MRT” and “Structural consistency of the diagram/pictogram”.

After the second evaluation round, the recommended modifications were implemented, resulting in the validation of previously inadequate items. Consequently, the MRT-EOPS was deemed adequate, leading to the acceptance of the alternative hypothesis.

## Data Availability

The research data are available only upon request.
