# Supplementary material for: Evaluation of the middle-range theory of occupational stress in healthcare professionals
Source: Rev Bras Enferm. 2025 Nov 3;78(4):e20240505. doi: 10.1590/0034-7167-2024-0505 (PMC12584948; doi:10.1590/0034-7167-2024-0505)
Supplement: Translation

# Translation: Avaliação da teoria de médio alcance do estresse ocupacional em profissionais de saúde

**DOI:** 10.1590/0034-7167-2024-0505pt

**Authors:** Romanniny Hévillyn Silva Costa Almino, Richardson Augusto Rosendo da Silva, Larissa Lilian Costa Firmino Segundo, Yasmim Yngrid Fernandes de Freitas, Vinicius dos Santos Lemos Pereira, Isabele Silva dos Santos, Thais Targino Ferreira, Roberta Keile Gomes de Sousa Manso

**Affiliations:** I Empresa Brazileira de Serviços Hospitalares. Natal, Rio Grande do Norte, Brasil; II Instituto Federal do Rio Grande do Norte. Natal, Rio Grande do Norte, Brasil; III Universidade Federal do Rio Grande do Norte. Natal, Rio Grande do Norte, Brasil

**Keywords:** Estresse Ocupacional, Pessoal de Saúde, Enfermagem, Estudos de Validação, Teoria de Enfermagem

## Abstract

**Objetivos:**

avaliar a Teoria de Médio Alcance do Estresse Ocupacional em Profissionais de Saúde com base na opinião de juízes.

**Métodos:**

trata-se de um estudo metodológico. Realizou-se a avaliação da referida teoria a partir dos critérios de Fawcett e utilizaram-se as fases descritas por Borel et al. para avaliação de teorias de enfermagem.

**Resultados:**

a avaliação ocorreu em duas rodadas. A amostra correspondeu a cinco juízes. Na primeira rodada, a teoria foi considerada adequada quanto à significância, parcimônia, testabilidade e adequação empírica. Contudo, apenas um item relacionado à consistência interna e um item relacionado à adequação pragmática não foram considerados adequados. Já na segunda rodada, os itens não validados anteriormente foram considerados adequados.

**Conclusões:**

a Teoria de Médio Alcance do Estresse Ocupacional em Profissionais de Saúde foi avaliada como adequada, sendo aceita a hipótese alternativa. A teoria permitirá a identificação dos estressores, dos fatores de proteção relacionados ao estresse ocupacional e a realização de intervenções pelo enfermeiro, demonstrando o impacto do estudo.

## INTRODUÇÃO

O desenvolvimento de teorias de enfermagem desempenha um papel fundamental no avanço da prática, da pesquisa e da educação na área. Essas teorias fornecem uma estrutura essencial para a prática profissional, orientando a tomada de decisões e contribuindo para a melhoria da qualidade do cuidado prestado aos pacientes^(1)^. Além disso, possibilitam uma compreensão mais aprofundada dos fenômenos relacionados à saúde e ao cuidado, facilitando a comunicação entre os profissionais e promovendo a padronização das práticas^(2)^.

Dentre as diferentes abordagens teóricas, as teorias de médio alcance (TMA) destacam-se pela sua aplicabilidade no cuidado em saúde. Diferentemente das grandes teorias, que são mais abstratas e abrangentes, as TMA são mais específicas e focadas em aspectos particulares do cuidado, tornando-se altamente úteis na prática clínica diária^(3)^. Um exemplo significativo de sua aplicação ocorre no cuidado à saúde do trabalhador, especialmente entre os profissionais da área da saúde

Esses profissionais estão constantemente expostos a diversos estressores no ambiente de trabalho, incluindo jornadas exaustivas, alta demanda emocional e risco de exposição a doenças infecciosas. Esses fatores podem resultar em altos níveis de estresse, burnout e comprometimento da saúde mental e física^(4)^. Assim, a atenção à saúde desses trabalhadores é crucial, não apenas para preservar o seu bem-estar, mas também para garantir a qualidade do cuidado oferecido aos pacientes.

A aplicação das teorias de enfermagem no cuidado à saúde dos trabalhadores permite compreender e abordar os fatores que impactam seu bem-estar, como o estresse ocupacional^(5)^. Nesse contexto, várias teorias foram desenvolvidas, entre elas o Modelo de Sistemas de Betty Neuman, que pode originar uma TMA a partir de um método dedutivo. De acordo com esse modelo, o paciente (cliente) mantém uma interação recíproca constante com o ambiente, estando sujeito a estressores que podem comprometer a estabilidade do sistema e desencadear reações ao estresse, como o desenvolvimento de doenças. Dessa forma, o enfermeiro desempenha um papel fundamental ao implementar intervenções estratégicas para restaurar o equilíbrio do sistema do cliente^(6)^.

Um estudo propôs o diagnóstico de enfermagem Estresse Ocupacional, desenvolvido a partir da Teoria de Médio Alcance do Estresse Ocupacional em Profissionais de Saúde (TMA-EOPS)^(7)^, fundamentado em uma análise conceitual baseada no arcabouço teórico de Walker e Avant^(8)^ e operacionalizado por meio de uma revisão de escopo derivada do Modelo de Sistemas de Betty Neuman^(6)^.

A TMA-EOPS foi elaborada de forma dedutiva a partir do Modelo de Sistemas de Betty Neuman, incorporando conceitos-chave como sistema, cliente, estressores intra, inter e extrapessoais, instabilidade do sistema do cliente, doença, reações, variáveis de interação, estabilidade do sistema do cliente, bem-estar/saúde, enfermagem e prevenção primária, secundária e terciária. Essa teoria posiciona o profissional de saúde no centro, considerando sua vulnerabilidade aos estressores intra, inter e extrapessoais, bem como aos fatores de proteção. Caso o profissional de saúde apresente respostas negativas aos estressores, poderá manifestar instabilidade, refletida em sintomas fisiológicos e psicológicos, o que, consequentemente, leva ao desenvolvimento do estresse ocupacional^(7)^.

Além disso, a avaliação de teorias permite um julgamento acerca da relevância e da adequação dessas construções teóricas à pesquisa, ao ensino, à assistência e à gestão em enfermagem^(9,10)^, justificando, assim, a realização do presente estudo.

Diante desse cenário e considerando a importância de avaliar a teoria desenvolvida, surge a seguinte questão de pesquisa: a TMA-EOPS atende aos critérios de significância, consistência interna, parcimônia, testabilidade, adequação empírica e pragmática?

## OBJETIVOS

Avaliar a Teoria de Médio Alcance do Estresse Ocupacional em Profissionais de Saúde (TMA-EOPS) com base na opinião de juízes, a partir dos critérios de Fawcett^(11)^ e das fases descritas por Borel et al^(12)^.

## MÉTODOS

### Aspectos éticos

Esta pesquisa seguiu os preceitos éticos estabelecidos pela Resolução 466/12 do Conselho Nacional de Saúde, tendo sido aprovada pelo Comitê de Ética em Pesquisa.

### Desenho, período e local do estudo

Trata-se de um estudo metodológico voltado à avaliação da Teoria de Médio Alcance do Estresse Ocupacional em Profissionais de Saúde (TMA-EOPS). Para sua execução, empregou-se o método Delphi, fundamentado nos critérios de Fawcett^(11)^. O método Delphi é amplamente utilizado para obter consenso coletivo sobre determinado tema avaliado por um grupo de especialistas^(12,13)^.

Neste estudo, visando à avaliação de uma teoria de enfermagem, foram seguidas as fases descritas por Borel et al^(12)^: fase preparatória, fase intermediária e fase de avaliação da teoria. A etapa de avaliação foi realizada no período de junho a novembro de 2022.

A fase preparatória consistiu na definição da teoria a ser analisada e na escolha dos critérios de avaliação. Assim, a TMA-EOPS foi avaliada com base nos seguintes critérios propostos por Fawcett^(11)^: significância, consistência interna, parcimônia, testabilidade, adequação empírica e pragmática.

Apenas um item foi adaptado na consistência interna, com a inclusão do seguinte ponto de avaliação: “o diagrama/pictograma apresenta consistência estrutural”. Além disso, esta pesquisa foi estruturada conforme as diretrizes do *Revised Standards for Quality Improvement Reporting Excellence* (SQUIRE 2.0)^(14)^.

### População ou amostra; critérios de inclusão e exclusão

Para seleção dos juízes foi realizada uma busca da Plataforma Lattes do Conselho Nacional de Desenvolvimento Científico e Tecnológico (CNPq) mediante emprego das seguintes palavras-chave: enfermagem AND teoria de enfermagem AND estudo de validação.

Posteriormente, realizou-se a categorização dos avaliadores/juízes da TMA-EOPS, de acordo com os critérios proposto por Borel et al^(12)^ quanto à formação educacional, experiência profissional, conhecimento metateórico e difusão de conhecimento produzido e reconhecimento da *expertise* no tema de metateorias ou teorias de enfermagem.

Salienta-se que para o item “experiência profissional na área da teoria a ser avaliada” se considerou a experiência geral em teorias de enfermagem, já que a TMA-EOPS ainda estava em fase de desenvolvimento.

Os avaliadores/juízes foram convidados, por e-mail, mediante carta convite contendo o Termo de Consentimento Livre e Esclarecido (TCLE) com as informações relacionadas ao estudo e orientações para preenchimento do instrumento. A obtenção dos e-mails ocorreu a partir da plataforma lattes, sites da instituição de vinculação dos juízes ou por outras páginas da internet. Estipulou-se como prazo inicial 20 dias para retorno do preenchimento da pesquisa para aqueles que aceitaram participar do estudo. Os juízes selecionados foram recordados da pesquisa a cada 15 dias.

### Protocolo do estudo

A seguir, detalham-se os nove pontos que compõem a fase intermediária, conforme Borel et al^(12)^:

1) Definição do tipo de Delphi: Utilizou-se um instrumento de avaliação baseado em escala Likert, com espaços para sugestões e comentários dos avaliadores.
2) Funções do coordenador, avaliador primário e secundário: O coordenador/avaliador primário foi responsável por fornecer aos avaliadores secundários o material necessário para a avaliação da TMA-EOPS^(12)^.
3) Seleção dos candidatos a avaliadores: Conforme Borel et al^(12)^, os juízes foram classificados segundo o nível de perícia presumida, categorizados como iniciante, iniciante avançado, competente, proficiente ou perito. Destaca-se que o tamanho da amostra depende do nível de expertise dos avaliadores.
4) Convite aos avaliadores: Como mencionado anteriormente, o convite foi realizado via e-mail, com envio do TCLE.

Na Figura, encontra-se o fluxograma de recrutamento dos juízes para a avaliação da TMA-EOPS.

**Figura 1:** Fluxograma do processo de recrutamento dos juízes para a avaliação da Teoria de Médio Alcance do Estresse Ocupacional em Profissionais de Saúde (TMA-EOPS)

5) Reaplicação dos critérios de categorização dos avaliadores pelo nível de perícia e ajustes na composição das equipes de juízes: Após a categorização inicial, não houve necessidade de adaptações na composição das equipes de avaliadores.
6) Planejamento de rodadas e critérios de interrupção: A quantidade de rodadas foi definida com base no alcance do consenso entre os juízes. Foram realizadas duas rodadas de avaliação, e o limite de corte mínimo para atingir o nível de concordância estabelecido neste estudo foi de 0,8 ou 80%^(15)^.
7) Estabelecimento de critérios explícitos para descarte de itens em cada rodada: Os itens que não atingiram o corte mínimo para concordância foram excluídos das análises subsequentes.
8) Definição do alcance do consenso e estabilidade das respostas dos juízes: A avaliação dos itens foi conduzida por meio de uma escala Likert de cinco pontos, com as seguintes opções: (1- discordo totalmente, 2- discordo parcialmente, 3- não discorda nem concorda, 4-concordo parcialmente, 5-concordo totalmente). Para a análise dos dados, todas as variáveis medidas por escala Likert foram agrupadas em três categorias: 1 e 2 (discorda), 3 (indiferente), 4 e 5 (concorda).
9) Fornecimento de orientações sobre a estratégia de avaliação teórica aos juízes: Os juízes receberam instruções detalhadas acerca dos critérios de avaliação teórica, garantindo maior clareza e uniformidade na análise dos itens propostos.

### Análise dos resultados e estatística

Para a execução dos métodos propostos, foram utilizados o Microsoft Excel 2019 e o software R, versão 4.2.2. Inicialmente, realizou-se uma análise descritiva e inferencial dos dados. Os testes estatísticos empregados neste estudo foram: Índice de Validade de Conteúdo (IVC), Coeficiente V de Aiken e Kappa multi-avaliador marginal livre.

Além disso, foram estabelecidas as seguintes hipóteses estatísticas relacionadas à avaliação da Teoria de Médio Alcance do Estresse Ocupacional em Profissionais de Saúde (TMA-EOPS): Hipótese nula (H₀): Os itens da TMA-EOPS avaliados como adequados apresentam um índice inferior a 80%. Hipótese alternativa (H₁): Os itens da TMA-EOPS avaliados como adequados apresentam um índice igual ou superior a 80%.

## RESULTADOS

Observou-se que a maior parte dos juízes era do sexo feminino (60%), possuía título de doutorado (80%) e atuava como docente (80%). Além disso, 40% dos avaliadores foram classificados como peritos, demonstrando um alto nível de especialização na área. Ademais, 80% dos participantes desenvolviam atividades relacionadas à orientação e estudos sobre teorias de enfermagem.

Com relação ao Índice de Validação de Conteúdo (IVC), ao Coeficiente V de Aiken e ao Kappa multi-avaliador marginal livre, verificou-se que apenas o item “Há possibilidade de comparação dos resultados obtidos com a implementação das intervenções de enfermagem oriundas da TMA e sem a utilização da teoria” não atingiu o valor crítico de 0,8, tornando-se necessária sua readequação, conforme Tabela 1.

**Tabela 1:** Avaliação pelos juízes, na segunda rodada, da Teoria de Médio Alcance do Estresse Ocupacional em Profissionais de Saúde, segundo critérios de Fawcett, conforme IVC, coeficiente de Aiken e Kappa multiavaliador marginal livre, Natal, Rio Grande do Norte, Brasil, 2022

CRITÉRIO	ITEM	IVC	Coeficiente de Aiken	Kappa
Significância	Os conceitos e proposições da TMA são explícitos, incluindo os conceitos metaparadigmáticos.	1,00	1,00	1,00
	As afirmações filosóficas nas quais a teoria se baseia são explícitas.	1,00	1,00	1,00
	O modelo conceitual do qual a TMA foi derivada é explícito.	1,00	1,00	1,00
Consistência interna	Há congruência do contexto (aspectos filosóficos e modelo conceitual) e do conteúdo (conceitos e proposições) da TMA.	1,00	1,00	1,00
	Os conceitos apresentam clareza e consistência semântica.	1,00	1,00	1,00
	As proposições apresentam consistência semântica.	1,00	1,00	1,00
	As proposições apresentam consistência estrutural.	1,00	1,00	1,00
	O diagrama/pictograma apresenta consistência estrutural.	1,00	1,00	1,00
Parcimônia	O conteúdo teórico é declarado de forma clara e concisa.	1,00	1,00	1,00
Testabilidade	A metodologia da pesquisa reflete a Teoria de Médio Alcance.	1,00	1,00	1,00
	Os conceitos da Teoria de Médio Alcance são observáveis por meio de indicadores empíricos apropriados.	1,00	1,00	1,00
	As proposições teóricas são passíveis de testes empíricos.	1,00	1,00	1,00
Adequação empírica	As afirmações teóricas são congruentes com as evidências empíricas.	1,00	1,00	1,00
Adequação pragmática	O ensino da TMA é necessário antes da aplicação da teoria na prática de enfermagem.	1,00	1,00	1,00
	A derivação da TMA é viável.	1,00	1,00	1,00
	O enfermeiro tem a capacidade legal para implementar e medir a eficácia da teoria baseada nas intervenções de enfermagem.	1,00	1,00	1,00
	As intervenções de enfermagem baseadas na TMA são compatíveis de serem implementadas na prática de enfermagem.	1,00	1,00	1,00
	As intervenções de enfermagem baseadas na TMA poderão conduzir ao alcance de resultados favoráveis.	1,00	1,00	1,00
	Há possibilidade de comparação dos resultados obtidos com a implementação das intervenções de enfermagem oriundas da TMA e sem a utilização da teoria.	1,00	1,00	1,00
	Os resultados poderão ser mensurados em termos de eficácia da TMA para resolução dos problemas.	1,00	1,00	1,00
	Avaliação global			1,00

*TMA – Teoria de Médio Alcance; IVC – índice de validação de conteúdo.*

Além disso, o item “O diagrama/pictograma apresenta consistência estrutural” obteve um índice abaixo de 0,8 no teste de Kappa multi-avaliador marginal livre, realizado na primeira rodada, sendo recomendada a melhoria desse aspecto na estrutura teórica.

Após as alterações sugeridas pelos juízes na segunda rodada de avaliação, o item “Há possibilidade de comparação dos resultados obtidos com a implementação das intervenções de enfermagem oriundas da TMA e sem a utilização da teoria” obteve a pontuação máxima nos seguintes indicadores: IVC, Coeficiente V de Aiken e Kappa multi-avaliador marginal livre. Os resultados detalhados encontram-se na Tabela 2.

**Tabela 2:** Avaliação pelos juízes, na segunda rodada, da Teoria de Médio Alcance Estresse Ocupacional em Profissionais de Saúde, segundo critérios de Fawcett, conforme IVC, coeficiente de Aiken e kappa multi avaliador marginal livre, Natal, Rio Grande do Norte, Brasil, 2022

CRITÉRIO	ITEM	IVC	Coeficiente de Aiken	Kappa
Significância	Os conceitos e proposições da TMA são explícitos, incluindo os conceitos metaparadigmáticos.	1,00	1,00	1,00
	As afirmações filosóficas nas quais a teoria se baseia são explícitas.	1,00	1,00	1,00
	O modelo conceitual do qual a TMA foi derivada é explícito.	1,00	1,00	1,00
Consistência interna	Há congruência do contexto (aspectos filosóficos e modelo conceitual) e do conteúdo (conceitos e proposições) da TMA.	1,00	1,00	1,00
	Os conceitos apresentam clareza e consistência semântica.	1,00	1,00	1,00
	As proposições apresentam consistência semântica.	1,00	1,00	1,00
	As proposições apresentam consistência estrutural.	1,00	1,00	1,00
	O diagrama/pictograma apresenta consistência estrutural.	1,00	1,00	1,00
Parcimônia	O conteúdo teórico é declarado de forma clara e concisa.	1,00	1,00	1,00
Testabilidade	A metodologia da pesquisa reflete a Teoria de Médio Alcance.	1,00	1,00	1,00
	Os conceitos da Teoria de Médio Alcance são observáveis por meio de indicadores empíricos apropriados.	1,00	1,00	1,00
	As proposições teóricas são passíveis de testes empíricos.	1,00	1,00	1,00
Adequação empírica	As afirmações teóricas são congruentes com as evidências empíricas.	1,00	1,00	1,00
Adequação pragmática	O ensino da TMA é necessário antes da aplicação da teoria na prática de enfermagem.	1,00	1,00	1,00
	A derivação da TMA é viável.	1,00	1,00	1,00
	O enfermeiro tem a capacidade legal para implementar e medir a eficácia da teoria baseada nas intervenções de enfermagem.	1,00	1,00	1,00
	As intervenções de enfermagem baseadas na TMA são compatíveis de serem implementadas na prática de enfermagem.	1,00	1,00	1,00
	As intervenções de enfermagem baseadas na TMA poderão conduzir ao alcance de resultados favoráveis.	1,00	1,00	1,00
	Há possibilidade de comparação dos resultados obtidos com a implementação das intervenções de enfermagem oriundas da TMA e sem a utilização da teoria.	1,00	1,00	1,00
	Os resultados poderão ser mensurados em termos de eficácia da TMA para resolução dos problemas.	1,00	1,00	1,00
	Avaliação global			1,00

TMA – Teoria de Médio Alcance; IVC – índice de validação de conteúdo.

A partir das sugestões dos juízes na avaliação, identificou-se a necessidade de realizar ajustes no diagrama da Teoria de Médio Alcance do Estresse Ocupacional em Profissionais de Saúde (TMA-EOPS). As principais recomendações foram:

- Correção do sentido das setas dos estressores, que anteriormente apontavam para a estabilidade do processo saúde-doença, o que exigiu um ajuste na direção dos vetores para melhor representar a influência dos fatores estressores.
- Adequação das cores do diagrama, visando maior clareza na distinção dos conceitos relacionados ao alívio e aparecimento do estresse ocupacional.
- Expansão da explicação sobre a utilidade da teoria, conforme sugestão de um dos avaliadores, para reforçar sua aplicação prática e relevância científica.

Diante dessas observações, foram implementadas adequações no diagrama fundamentadas na cromoterapia, conforme descrito a seguir:

- As setas partem do centro do esquema, simbolizando a resposta do profissional de saúde frente à influência dos estressores e dos fatores de proteção no seu processo saúde-doença.
- Azul: Representa os conceitos relacionados à estabilidade do processo saúde-doença, incluindo saúde e fatores de proteção.
- Vermelho: Indica os elementos ligados à instabilidade do processo saúde-doença, abrangendo estressores, sintomas fisiológicos e psicológicos e o estresse ocupacional.
- Verde: Refere-se aos conceitos que visam manter a estabilidade do processo saúde-doença, como enfermagem, intervenções de enfermagem, promoção da saúde, prevenção secundária e terciária.

As alterações implementadas estão representadas na Figura 2.

**Figura 2:** Diagrama da Teoria de Médio Alcance do Estresse Ocupacional em Profissionais de saúde (TMA/EOPS)

Portanto, após alterações sugeridas pelos juízes na segunda rodada de avaliação, o item “o diagrama/pictograma apresenta consistência estrutural” apresentou a pontuação máxima para o IVC, coeficiente de Aiken e Kappa multi avaliador.

Assim, como o IVC de todos os itens avaliados foram > que 0,8, o valor crítico de Aiken ≥ 0,8, e/ou Kappa multi-avaliador marginal livre >0,8, a hipótese alternativa foi aceita no estudo.

Considerando que a Teoria de Médio Alcance do Estresse Ocupacional em Profissionais de Saúde (TMA-EOPS) foi desenvolvida recentemente, ainda não houve possibilidade de comparar os dados de implementação das intervenções de enfermagem oriundas da teoria com aqueles obtidos sem sua utilização.

Diante dessa limitação, destacou-se a importância de realizar a testagem da TMA-EOPS, a fim de avaliar sua credibilidade científica e sua aplicabilidade prática. Para isso, recomenda-se o desenvolvimento de pesquisas com diferentes delineamentos metodológicos, incluindo estudos qualitativos, quantitativos e de métodos mistos, que possam testar a teoria em diversas áreas de atuação do enfermeiro, tais como assistência, docência, pesquisa e gerência.

Após as alterações sugeridas pelos juízes na segunda rodada de avaliação, verificou-se que o item “O diagrama/pictograma apresenta consistência estrutural” obteve pontuação máxima nos seguintes indicadores: IVC, Coeficiente V de Aiken e Kappa multi-avaliador marginal livre.

Além disso, como o IVC de todos os itens avaliados apresentou valores superiores a 0,8, e o valor crítico do coeficiente de Aiken foi ≥ 0,8, assim como o Kappa multi-avaliador marginal livre obteve resultados acima de 0,8, a hipótese alternativa foi aceita no estudo.

## DISCUSSÃO

As teorias de enfermagem oferecem modelos assistenciais que aprimoram a atuação do enfermeiro na prática clínica^(16)^. Em outras palavras, fundamentam o conhecimento científico e contribuem para o refinamento das atividades profissionais, por meio do planejamento e da organização dos processos assistenciais.

Nesse contexto, as Teorias de Médio Alcance (TMA) representam métodos eficazes para aproximar teoria e prática, permitindo que sejam utilizadas como referências estruturantes na prática clínica de enfermagem^(17)^. A avaliação das TMA, quando ainda em processo de desenvolvimento, possibilita a determinação do nível de relevância da teoria para as diversas áreas da enfermagem, além de verificar se atende aos critérios estabelecidos para avaliação^(10,11)^.

O Método de Fawcett compreende um conjunto de critérios específicos que buscam avaliar as teorias de enfermagem a partir da análise de significância, consistência interna, parcimônia, testabilidade, adequação empírica e pragmática^(11)^. Dessa forma, o Método de Fawcett foi selecionado e empregado como estratégia para examinar a relevância da TMA-EOPS na fase preparatória da Teoria de Médio Alcance, seguindo o método Delphi, conforme as diretrizes propostas por Borel et al^(12)^.

Os critérios estabelecidos por Borel et al^(12)^ foram empregados na seleção dos juízes responsáveis pela avaliação da Teoria de Médio Alcance do Estresse Ocupacional em Profissionais de Saúde (TMA-EOPS), considerando aspectos essenciais para classificar o nível de perícia dos avaliadores. Esses critérios incluem: formação educacional, experiência profissional, conhecimento metateórico, difusão do conhecimento produzido na área e reconhecimento da expertise em metateorias ou teorias de enfermagem.

A TMA-EOPS, desenvolvida neste estudo, foi avaliada pelos juízes por meio do método Delphi, baseado na sabedoria coletiva. A avaliação de teorias desempenha um papel essencial no desenvolvimento do conhecimento em enfermagem, pois possibilita o aprimoramento de seus elementos, refinando-os antes de sua disseminação na prática e após sua aplicação^(10,12)^.

Na primeira etapa de avaliação da TMA-EOPS, os seguintes critérios foram considerados adequados pelos juízes: significância, parcimônia, testabilidade e adequação empírica.

Segundo Fawcett^(11)^, a significância de uma teoria abrange suas origens metaparadigmáticas, filosóficas e os modelos conceituais dos quais foi derivada, sendo essencial que tais informações sejam explicitadas com clareza.

A TMA-EOPS foi derivada do modelo teórico de Betty Neuman, o qual adota uma perspectiva filosófica holística e sofre influência da Teoria Geral dos Sistemas, apresentando relações diretas com os conceitos da Gestalt e do estresse. Além disso, a teoria é voltada para a promoção do bem-estar^(7)^.

A parcimônia refere-se à avaliação do conteúdo da teoria, priorizando a simplicidade e a objetividade. Uma teoria bem estruturada deve apresentar um número reduzido de conceitos e proposições, sem comprometer sua capacidade explicativa, de modo que seja suficientemente clara para elucidar o fenômeno de interesse^(11)^. No caso da TMA-EOPS, foram definidos 16 conceitos principais e 20 proposições^(7)^.

O critério da testabilidade, por sua vez, está relacionado tanto ao conteúdo da teoria quanto à sua aplicabilidade prática. Para que uma Teoria de Médio Alcance (TMA) seja considerada testável, seus conceitos devem possuir definições operacionais, enquanto suas proposições devem ser passíveis de observação e/ou mensuração^(11)^. No contexto da TMA-EOPS, foram descritas as definições dos estressores e sintomas abordados na teoria^(7)^.

Segundo Fawcett^(11)^, a adequação empírica é verificada quando as declarações contidas na teoria estão fundamentadas em evidências científicas. No caso da TMA-EOPS, todos os conceitos e proposições foram originados a partir dos achados das 138 publicações que compuseram a Scoping Review^(7)^.

Por fim, a consistência interna corresponde à clareza e coerência semântica da teoria. Dessa forma, todos os conceitos, proposições e afirmações devem apresentar congruência estrutural e lógica^(11)^. Durante a avaliação da TMA-EOPS, observou-se que sua representação gráfica inicial não apresentava total consistência, entretanto, após as adequações sugeridas pelos juízes, esse aspecto foi corrigido e aprimorado.

A apresentação do diagrama da TMA-EOPS reafirma a compreensão da teoria, evidenciando seus dois componentes centrais: o estresse e a reação ao estresse. Esses elementos são dispostos de maneira clara e estruturada no modelo visual, permitindo uma melhor assimilação dos conceitos pelos profissionais de saúde^(18)^.

O diagrama da TMA-EOPS incorpora princípios da cromoterapia, ciência utilizada na área da saúde. As cores empregadas possuem significados terapêuticos, sendo utilizadas para representar diferentes aspectos da teoria. A cor azul tem papel importante na redução do estresse, já a cor verde auxilia na redução do estresse e simboliza a área da saúde. Por sua vez, a cor vermelha estar relacionada ao estresse^(19)^.

Ao alinhar teoria e prática clínica, a TMA-EOPS proporciona benefícios significativos para os campos do ensino e da pesquisa, contribuindo para uma maior integração entre a prática profissional e o conhecimento acadêmico. Dessa forma, a teoria fortalece a base científica da enfermagem em saúde ocupacional, fornecendo diretrizes substanciais para a atuação dos enfermeiros.

Além disso, a gestão em saúde também é contemplada, pois a teoria ressalta a importância da colaboração interdisciplinar na elaboração de estratégias eficazes para a prevenção do estresse ocupacional.

As aplicações práticas da teoria estão relacionadas à identificação dos estressores intra, inter e extrapessoais, bem como dos fatores de proteção contra o estresse ocupacional. A TMA-EOPS também orienta a implementação de intervenções preventivas, podendo atuar em níveis primário, secundário e terciário.

Por fim, a TMA-EOPS adota uma abordagem filosófica holística, sendo capaz de explicar de forma consistente o fenômeno do estresse ocupacional em profissionais de saúde, fornecendo subsídios para ações estratégicas e preventivas no contexto clínico.

### Limitações do estudo

Considerando que a TMA-EOPS foi desenvolvida recentemente, ainda não houve possibilidade de comparar os dados de sua implementação, tampouco os resultados das intervenções de enfermagem derivadas da teoria, configurando-se como uma limitação do estudo.

Dessa forma, destaca-se a importância de validações futuras no contexto clínico, permitindo a aplicação prática da teoria e a verificação de sua efetividade. Para tanto, recomenda-se o desenvolvimento de pesquisas com diferentes delineamentos metodológicos, incluindo abordagens qualitativas, quantitativas e de métodos mistos, a fim de testar a TMA-EOPS nas diversas áreas de atuação do enfermeiro: assistência, docência, pesquisa e gerência.

### Contribuições do estudo para a área da Enfermagem, Saúde ou Política Pública

A TMA-EOPS apresenta-se como uma ferramenta promissora para a prática clínica dos enfermeiros que atuam na área de saúde do trabalhador, uma vez que se fundamenta em uma abordagem baseada em evidências.

Além disso, permite a identificação dos estressores e dos fatores de proteção associados ao estresse ocupacional, possibilitando a implementação de intervenções eficazes voltadas à promoção da saúde mental dos trabalhadores.

## CONCLUSÕES

O estudo permitiu a avaliação da Teoria de Médio Alcance do Estresse Ocupacional em Profissionais de Saúde (TMA-EOPS) com base nos critérios de Fawcett, por meio da aplicação do método Delphi, conforme as fases descritas por Borel et al.

Na primeira rodada de avaliação, dois itens apresentaram índices inferiores ao esperado, tornando necessária a realização de ajustes e revisões: “Possibilidade de comparação dos resultados obtidos com e sem a implementação das intervenções de enfermagem baseadas na TMA” e “Consistência estrutural do diagrama/pictograma”.

Após a segunda rodada, as modificações recomendadas pelos juízes foram implementadas, resultando na validação dos itens anteriormente considerados inadequados. Dessa forma, a TMA-EOPS foi avaliada como adequada, levando à aceitação da hipótese alternativa.

## Data Availability

Os dados de pesquisa só estão disponíveis mediante solicitação.
